# The Association Between Diabetes Mellitus and Risk of Sarcopenia: Accumulated Evidences From Observational Studies

**DOI:** 10.3389/fendo.2021.782391

**Published:** 2021-12-23

**Authors:** Yu-Shun Qiao, Yin-He Chai, Hong-Jian Gong, Zhiyessova Zhuldyz, Coen D. A. Stehouwer, Jian-Bo Zhou, Rafael Simó

**Affiliations:** ^1^ Beijing Tongren Hospital, Capital Medical University, Beijing, China; ^2^ Department of Internal Medicine and Cardiovascular Research Institute Maastricht (CARIM) School for Cardiovascular Diseases, Maastricht University Medical Center, Maastricht, Netherlands; ^3^ Department of Endocrinology, Beijing Tongren Hospital, Capital Medical University, Beijing, China; ^4^ Endocrinology and Nutrition Derpartment, Vall d’Hebron University Hospital, Autonomous University, Barcelona, Spain; ^5^ Diabetes and Metabolism Research Unit, Vall d’Hebron Research Institute (VHIR), Barcelona, Spain; ^6^ Centro de Investigación Biomédica en Red de Diabetes y Enfermedades Metabólicas Asociadas (CIBERDEM), Instituto de Salud Carlos III (ICSIII), Madrid, Spain

**Keywords:** sarcopenia, HbA1c, prediabetes, diabetic complications, diabetes mellitus, observational study

## Abstract

**Aim:**

We performed a meta-analysis of observational studies to evaluate the association between the presence of sarcopenia and HbA1c, prediabetes, diabetes and diabetic complications.

**Method:**

The PubMed, Embase, Cochrane and Web of Science databases were searched from inception to May 2021. We included full-text English language articles that reported the prevalence of sarcopenia in patients with and without diabetes. Quality assessment was performed according to the Newcastle- Ottawa scale for observational studies.

**Results:**

Sixteen studies were included in the meta-analysis. Three studies showed that high HbA1c levels lead to loss of muscle mass, and one study involving prediabetes showed that people with prediabetes had lower muscle mass, strength, and performance than non-diabetic population. Seven studies showed that people with diabetes had a higher risk of sarcopenia than those without diabetes (combined OR: 2.09, 95% CI:1.62-2.70). The remaining five studies suggested that diabetic complications increased the risk of sarcopenia (combined OR: 2.09,95% CI:1.62-2.70).

**Conclusion:**

High HbA1c levels, prediabetes, diabetes and diabetes complications were associated with an increased risk of sarcopenia. Therapeutic strategies addressed to avoid the conversion of IGT to diabetes and to optimize glycemic control are warranted to prevent or arrest sarcopenia in the diabetic population

## Introduction

The term sarcopenia refers to the loss of muscle mass, muscle strength or physical function that occurs with aging. Sarcopenia is an emerging health concern that mainly affects the quality of life of older people and is an underlying factor for falls, fractures, deaths and a series of adverse events (1–3). Therefore, early identification is crucial for implementing appropriate preventive actions and studying its pathogenesis and influencing factors, such as aging (4), obesity (5), cancer (6) and other diseases (7, 8). Diabetes has been reported as an influencing factor for sarcopenia (9). When insulin resistance occurs in skeletal muscles, glucose utilization and protein synthesis are reduced, which, in turn, aggravates insulin resistance and muscle loss, thus evolving into a vicious circle (10, 11). According to statistics from a systematic literature review, up to 70% of adults with diabetes have difficulty performing routine physical tasks, with lower extremity mobility limitations particularly evident (12).

Further, literature shows that many previous studies (13) have suggested that patients with diabetes have a significantly increased risk of sarcopenia. However, it is unknown whether this relationship is also present in subjects with prediabetes. In addition, the role of glycemic control and diabetic complications remains to be elucidated. Therefore, we conducted a meta-analysis of the literature to study the relationship between sarcopenia and diabetes, prediabetes, glycemic control and diabetic complications.

## Method

### Search Strategy

This meta-analysis followed the Preferred Reporting Items for Systematic Reviews and Meta-Analysis guidelines. We systematically searched for relevant literature in the PubMed, Embase, Cochrane and Web of Science databases for studies published from inception to May 2021. The following items (single or combined) were included in the search strategy: sarcopenia, skeletal muscle, muscle mass, muscle strength, physical performance, gait speed, hyposthenia, grip strength, diabetes, diabetes mellitus, hemoglobin a1c, impaired fasting glucose, impaired glucose tolerance and prediabetes. The studies were based on patients with type 2 diabetes mellitus (T2DM), rather than type 1 diabetes mellitus (T1DM) or other special types of diabetes. Each study was required to consider essential variables, such as age (years), sex (% male), duration of diabetes (years) and body mass index (kg/m²). The reference lists of all retrieved articles were manually reviewed. Two independent authors (YHC and independently) analyzed each article and performed data extraction. A third investigator consulted (xx) in cases of disagreement. Discrepancies were resolved by consensus.

### Diagnostic Criteria

This study only discussed the association between sarcopenia and T2DM rather than other types diabetes mellitus. T2DM was diagnosed as having fasting blood glucose measurement (FPG≥7.0 mmol/L) with or without 2-hour postprandial blood glucose ≥11.1 mmol/L during an oral glucose tolerance test (OGTT), according to World Health Organization (WHO) 1999 criteria. Prediabetes was defined as having 6.1 and 7.0mmol/L with or without a 2-hour OGTT between and 11.1mmol/L. The diagnostic criteria for sarcopenia and involved muscle mass measured by dual X-ray absorptiometry (DXA) or bioelectrical impedance analysis (BIA), in accordance with Asian Working Group for Sarcopenia (AWGS), European Working Group on Sarcopenia in Older People (EWGSOP), or Foundation for the NIH (FNIH) diagnostic criteria.

### Study Selection

The inclusion criteria for the selected articles were as follows: (1) the included study subjects were older than 60 years; (2) the prevalence of sarcopenia in the diabetic, prediabetic, and non-diabetic groups was calculated; (3) type of diabetes mellitus was T2DM; (4) low muscle mass was considered for the indispensable diagnosis of sarcopenia; (5) research design belonged to observational (cross-sectional or case–control or cohort) studies (6) extractable data was available on sarcopenia in adults with diabetes or prediabetes and euglycemic subjects.

The exclusion criteria for selected articles were as follows: (1) control group (euglycemic subjects) was not set up; (2) reviews, letters or conference abstracts; (3) repeat publications; (4) studies that were not written in English; (5) individuals with severe diabetes and complications who are unable to walk or lack activity capability; (6) participants with acute disease, history of stroke, myocardial infarction or cancer; (7) individuals aged >85 years, severe heart failure (New York Heart Association Class II-IV), or had severe liver impairment (liver enzyme ALT≥3-fold the upper limit of normal range), severe renal dysfunction (estimated glomerular filtration rate [eGFR]<30 mL/min/1.73m2), or a history of thyroid or adrenal diseases, were also excluded.

There were a few exceptions that should be explained here: (1) the study of Sambashivaish was on the association between prediabetes and sarcopenia. Hence, we did not limit our age to over 60 years. (2) while the mean age of participants was lesser (44.3 ± 9.4 years) in Anbalagan’s study, there was a age and sex match between the test group and control group. Therefore, the literature is involved in this study.

### Data Extraction and Quality Assessment

The following data were extracted: (1) first author (2) publication year (3) country (4) study design (5) the basic information of participants (total number, mean age and proportion of female) (6) the number of participants with diabetes, diabetes complications or prediabetes and control group subjects (7) the prevalence rate of sarcopenia. Quality assessment was conducted by using The Newcastle-Ottawa scale (NOS), including three aspects of assessment: selection of subjects, comparability and assessment of outcomes. NOS allows four stars for subject selection, two stars for comparability, and three stars for outcomes assessment, therefore, a study can get up to nine stars. The quality of each study was graded as low (0–3), moderate (4–6), or high (7–9). Two independent researchers respectively did quality assessments, and a third researcher rescored the different results. Differences were resolved by consensus.

### Statistical Analyses

We analyzed and pooled Odd Ratios and 95% Confidence Intervals of the association between sarcopenia and diabetes or diabetes complications from the raw prevalence data, respectively. The ORs of the relationship between sarcopenia and diabetes complications were calculated by adjusting for confounding factors and other diabetes complications, such as age, sex, and hypertension, to reduce the interaction between various diabetes complications. The ORs and 95%CIs were converted into the natural logarithm (ln OR) and standard error values. Heterogeneity testing was evaluated using the I² statistic, and a fixed effects model was applied when heterogeneity was small (I² ≤50%); otherwise, a random effects model was used. Funnel plots were used to assess the potential for publication bias. However, to avoid the possible impact of visual perception, Egger’s test was conducted to assess publication bias more accurately (P-values <0.1 indicated that there was no publication bias). To explore the source of heterogeneity, subgroup analyses were used to evaluate age (<60 and ≥60 years), types of diabetes vascular disease (microvascular and macrovascular), and types of diabetes complications (diabetic nephropathy, diabetic foot, diabetic retinopathy and diabetic neuropathy). A sensitivity analysis was used to evaluate the robustness of the meta-analysis results. All analyses were performed using Stata 12.0 software (StataCorp LP, TX). Statistical significance was set at P <0.05.

## Results

### Literature Search Outcomes and Validity Assessment

**Figure 1:**
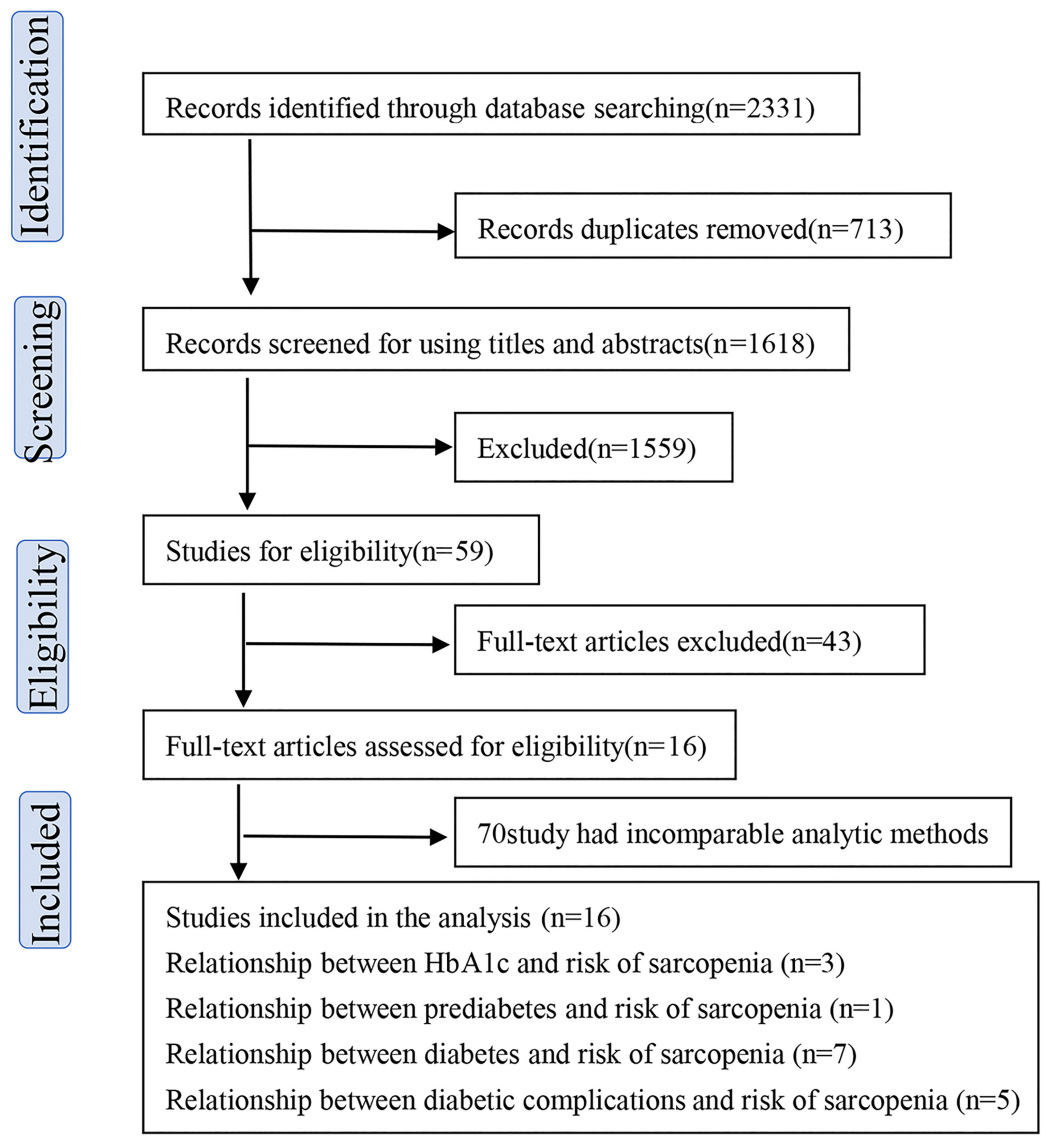
PRISMA (Preferred Reporting Items for Systematic Reviews and Meta-Analyses) study.

The search strategy identified 2,331 potentially relevant records, from multiple scientific studies and 713 were excluded as duplicates. The remaining manuscripts were searched for screening the title and abstract, and 1,559 publications were excluded because they were reviews, letters, or conference abstracts. Thus, 59 articles were eligible for full-text review and data assessment. Finally, 16 studies (14–29) were included in the current meta-analysis and systemic review. Among those publications, three (14–16) mentioned the relationship between HbA1c and sarcopenia, one (17) mentioned prediabetes, seven (18–24) mentioned diabetes, and five (25–29) mentioned diabetes-related complications, (**Figure 1**). The characteristics of studies included were shown in **Table 1**. The total sample size in 16 studies was 15,326, and all participants were >18 years old. All studies included control groups.

**Table 1:** Characteristics of the included studies.

ID	First author, Year of publication	Country	Study design	Mean age(years)	Diabetes duration (years)	HbA1c level (%)	Definition of sarcopenia	Sarcopenia diagnostic criteria	Method of measuring skeletal muscle mass	Diabetic complications	Therapy of diabetes	Participants(n)	Females (%)	Subjects with exposure factors (n)	Sarcopenia in subjects with exposure factors (n)	Subjects without exposure factors (n)	Sarcopeniain subjects without exposure factors(n)	Prevalence of sarcopenia in exposure factor (%)
1.	Sanada (14)	Japan	cross-sectional	65	NA	5	Low muscle mass	Self-definition	DXA	Not reported	NA	168	0	NA	NA	NA	NA	NA
2.	Sugimoto (15)	Japan	cross-sectional	68.7			Low muscle mass, strength and physical performance	AWGS	BIA		Insulin or oral diabetic medication	2813		NA	NA	NA	NA	NA
3.	Yoon (16)	Korea	Case-control	74.5			Low muscle mass, strength and physical performance	AWGS	DXA			269	0	NA	NA	NA	NA	NA
4.	Sambashivaish (17)	India	Case-control	39.2 ± 10.8	3.0 ± 1.5		Low muscle mass, strength	AWGS	DXA			169	0	44		125		
5.	Anbalagan (18)	India	Case-control	44.3 ± 9.4	4 ± 1.2		Low muscle mass	EWGSOP	DXA	no	Insulin or oral diabetic medication	152	44.7	76	30	76	12	39.5
6.	Bouchi (19)	Japan	Cross-sectional	64.8 ± 9.7		7.1	Low muscle mass, strength	AWGS	DXA			249	38.3	208	28	41	4	13.3
7.	Kim (20)	Korea	Case-control	70.5 ± 4.3	10.7 ± 8.8	8.2 ± 2.0	Low muscle mass	ASM/Ht^2^, ASM/Wt, TSM/Wt	DXA	no	Insulin or oral diabetic medication	189	0	59	34	130	54	57.6
8.	Kim (20)	Korea	Case-control	70.9 ± 5.4	13.0 ± 8.9	8.5 ± 2.3	Low muscle mass	ASM/Ht^2^, ASM/Wt, TSM/Wt	DXA	no	Insulin or oral diabetic medication	225	100	85	6	140	12	7.1
9.	Lim (21)	Korea	Cross-sectional	68.8 ± 8.2			Low muscle mass		DXA			3492	47	340	137			40.3
10.	Mori (22)	Japan	Cohort	58.1 ± 11.9	Not reported	Not reported	Low muscle mass, strength	AWGS	DXA			308	40	101	51	207	73	50.5
11.	Souza (23)	Brazil	Case-control	74.8 ± 11.4	Not reported		Low muscle mass, strength and physical performance	EWGSOP			Insulin or oral diabetic medication	1078	79.2	245	35	833	66	14.2
12.	Wang (24)	China	Cross-sectional	69.1 ± 7.2			Low muscle mass, strength and physical performance	AWGS	BIA		Insulin or oral diabetic medication	1090	52.3	236	35	854	96	14.8
13.	Celiker (25)	Turkey	Cross-Sectional	60.9 ± 6.9	10.0 ± 7.3		Low muscle mass, strength and physical performance	EWGSOP	BIA	Diabetic nephropathy	Not reported	103	64.8	50	17	53	8	34
14.	Cheng (26)	China	Cross-Sectional	64.6 ± 9.6	Not reported		Low muscle mass	AWGS	DXA	Diabetic foot	Not reported	1105	42.8	120	42	985	162	35.3
15.	Fukuda (27)	Japan	Cross-Sectional	63.4			Low muscle mass, strength	AWGS	DXA	Non-proliferative diabetic retinopathy	Insulin or oral diabetic medication	299	62.5	38		261		
16.	Fukuda (27)	Japan	Cross-Sectional	63.5			Low muscle mass, strength	AWGS	DXA	Proliferative diabetic retinopathy	Insulin or oral diabetic medication	278	62.2	17		261		
17.	Yang (28)	China	Cross-Sectional	64.7			Low muscle mass	Baumgartner diagnostic criteria	DXA	Diabetic foot	Not reported	1361	42	257	91	1104	204	35.4
18.	Yang (28)	China	Cross-Sectional	64.35 ± 9.32			Low muscle mass	Baumgartner diagnostic criteria	DXA	Diabetic neuropathy	Not reported	1104		796	163	308	41	20.5
19.	Yasemin (29)	Turkey	Cross-Sectional	60.2 ± 10.6			Low muscle mass, strength	EWGSOP	BIA	Diabetic neuropathy	Insulin or oral diabetic medication	602	59.5	512	127	90	8	24.7

NA, not applicable.

### Meta-Analysis of the Association Between Diabetes and the Risk of Sarcopenia

**Figure 2:**
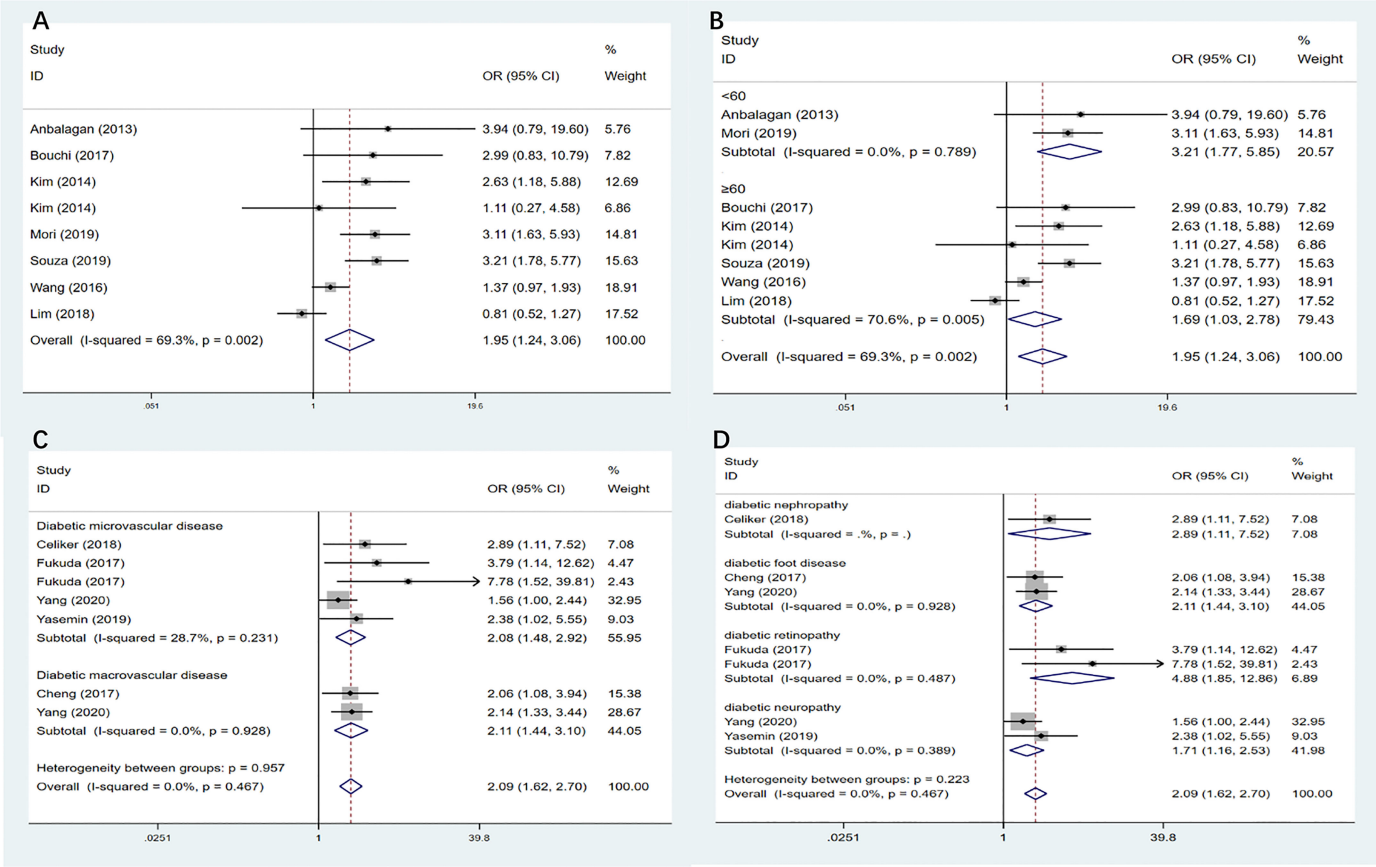
**(A)** Meta-analysis of the association between diabetes and the risk of sarcopenia. **(B)** Subgroup analysis of the association between diabetes and the risk of sarcopenia according to age. **(C)** Subgroup analysis of the association between diabetes complications and the risk of sarcopenia according to diabetic vascular disease. **(D)** Subgroup analysis of the association between diabetes complications and the risk of sarcopenia according to different diabetic complications. OR, odds ratio; CI, confidence interval. Where I^2^ is the variation in effect estimates attributable to heterogeneity, overall is the pooled random/fixed effect estimate of all studies. subtotal is the pooled random effects estimate of sub-group analysis studies. Weights are from random-effects analysis. %Weight is the weight assigned to each study, based on the inverse of the within- and between-study variance. The size of the grey boxes around the point estimates reflects the weight assigned to each study.

Seven studies (18–24) (n=6,783 participants, female: 52.7%) were included. The random effect model was used according to the I^2^. The combined OR was 1.95 (95% CI: 1.24–3.06, I²=69.3%, P=0.002), showing that patients with diabetes have a significantly higher risk of sarcopenia than patients without diabetes (**Figure 2A**).

The results of the subgroup analysis (**Figure 2B**) showed that the relationship between diabetes and the risk of sarcopenia was subject to age. Surprisingly, the risk of sarcopenia was higher in the patients aged <60 years (OR=3.21, 95% CI: 1.77—5.85, I²=0%) than in those aged ≥60 years (OR 1.69, 95% CI: 1.03—2.78, I²=70.6%). After removing studies one by one, the sensitivity analysis conclusively showed that patients with diabetes had a significantly higher risk of sarcopenia than patients without diabetes.

### Systemic Review of the Association Between HbA1c and the Risk of Sarcopenia

Three studies (14–16) mentioned the relationship between different HbA1c levels and the risk of sarcopenia. Unfortunately, we could not combine their data because of the different grouping standards. The first study (14) showed that men with class 1 sarcopenia (6.87< appendicular muscle mass (AMM)/height²<7.77 kg/m^2^) had higher levels of HbA1c than patients with normoglycemia. However, this association was not significant among women. In the second study (15), regardless sex, sarcopenia was positively correlated with HbA1c levels in subjects with T2DM. In addition, the authors reported that HbA1c levels were associated with low muscle mass rather than low muscle strength and performance. The third study (16) reported a negative relationship between HbA1c levels, muscle mass, and performance in patients with T2D, which was more evident when the HbA1c level was ≥8.5%. Overall, these studies confirmed the association between high HbA1c levels and sarcopenia.

### Systemic Review of the Association Between Prediabetes and the Risk of Sarcopenia

Only one study (17) focused on the association between prediabetes and the risk of sarcopenia. The participants in the study were all male, with ages ranging from 20 to 50 years. The study showed that subjects with prediabetes presented lower muscle mass, strength, and performance than non-diabetic subjects, suggesting that prediabetes is closely associated with the risk of sarcopenia.

### Meta-Analysis of the Association Between Diabetes Complications and the Risk of Sarcopenia

A total of five studies (25–29) (n=3286 participants, females: 46.4%) were included. The combined OR of the relationship between diabetes complications and the risk of sarcopenia was 2.09 (95% CI:1.62–2.70, I²=0%, P=0.467). This increased risk was maintained when microangiopathic and macroangiopathic complications were analyzed separately (**Figure 2C**, **Table 2**). The subgroup of diabetic complications (**Figure 2D**) showed that individuals with diabetic retinopathy had a higher significant risk than patients with diabetes without diabetic retinopathy, and the OR of diabetic retinopathy was 4.88 (95% CI:1.85—12.86). More importantly, proliferative diabetic retinopathy increased the risk of sarcopenia than non-proliferative diabetic retinopathy; the ORs were 7.78 (95% CI: 1.52—39.81) and 3.79 (95% CI: 1.14—12.62), respectively. Patients with diabetic nephropathy (OR=2.89, 95% CI: 1.11—7.52) or neuropathy (OR=1.71, 95% CI: 1.16—2.53) also had a higher prevalence of sarcopenia. After removing studies one by one, the sensitivity analysis suggests that patients with diabetic complications have a significantly higher risk of sarcopenia than patients with diabetes without complications.

**Table 2:** The OR values and 95%CI of diabetic complications.

Types of diabetic complications	OR value and 95%CI
Diabetic microangiopathic complications	2.08 (1.48-2.92)
Diabetic macroangiopathic complications	2.11 (1.44-3.10)
Diabetic nephropathy	2.89 (1.11-7.52)
Diabetic foot	2.11 (1.44-3.10)
Diabetic retinopathy	4.88 (1.85-12.86)
Diabetic neuropathy	1.71 (1.16-2.53)

### Quality Assessment

Quality assessment of the studies revealed scores of 6–9 on the NOS (30). Two studies scored 6, five were scored 7, seven were scored 8, and two were scored 9. Thus, high-quality studies were included in this meta-analysis (**Supplementary Table 1**).

### Publication Bias

According to the Cochrane Handbook (31), as a rule of thumb, tests for funnel plot asymmetry should be used only when there are enough studies, included in the meta-analysis because when there are fewer studies, the power of the tests is too low to distinguish real asymmetry from coincidence. In this study, the P-value of the Egger test was >0.05 (P = 0.227) for the relationship between diabetes and the risk of sarcopenia, indicating no significant bias. The funnel figure of these studies showed a symmetrical inverted distribution, which is consistent with the results of the Egger test (**Supplementary Figure 1**).

## Discussion

In this systematic review and meta-analysis, we included 16 studies to analyze the relationship between diabetes and sarcopenia. The results showed that the presence of diabetes, poor glycemic control, and related chronic complications significantly increased the risk of sarcopenia. In addition, a relationship between prediabetes and sarcopenia was observed.

Previous studies reported a harmful influence of diabetes on sarcopenia, which was based on studies showing that muscle mass, strength, and performance were significantly lower in patients with diabetes than in non-diabetic controls (32–35). However, few studies have examined whether there is a difference in the risk of sarcopenia among patients with diabetes, taking into account crucial variables such as the degree of glycemic control and the presence of long-term diabetic complications. Therefore, a novel contribution of this systematic review is its demonstration that poor glycemic control, assessed by HbA1c level, is an additional risk factor for the development or progression of sarcopenia among patients with diabetes. Although HbA1c reflects glycemic control in the previous 2–3 months (11), daily glycemic control is also very important. According to reports, large daily glycemic fluctuations also increased the risk of muscle loss (OR=1.045) (36), which suggests that both short-and long-term blood glycemic control should be emphasized.

Whether poor glycemic control is related to both muscle loss, muscle strength, and performance remains an open question. Three studies (14–16) using either body mass index, skeletal mass index (SMI), or appendicular skeletal muscle mass as measurements of muscle mass, showed that poor glycemic control was closely associated with the loss of muscle mass rather than the loss of muscle strength and performance. In contrast, in the Baltimore Longitudinal Study of Aging, knee extensor strength was lower across increasing quartiles of HbA1c (37). The differences in race, sample size, and assessment methods can explain the divergent results. Therefore, larger studies using uniform methods to evaluate muscle morphology and function are needed.

Muscle mass and prediabetes are likely to have bidirectional relationships. In a cross-sectional analysis of the National Health and Nutrition Examination Survey from the United States, after adjusting for multiple confounding factors, each 10% increase in SMI was associated with a 12% relative reduction in prediabetes prevalence (95% CI, 1%—21%) (38). These results suggest that muscle mass is inversely proportional to the risk of prediabetes. Since glucose levels are only slightly impaired in the prediabetic stage, it could be postulated that the pathways activated by insulin resistance, rather than those activated by chronic hyperglycemia, are meaningful in the development of sarcopenia.

A clear influence of diabetic complications was found on the risk of sarcopenia. In this regard, we would like to emphasize that diabetic retinopathy and diabetic nephropathy tend to coexist and can synergistically increase the risk of sarcopenia. In addition, although previous studies (39–41) have mostly analyzed the association between one or two complications of diabetes and sarcopenia, compared with patients without diabetic complications, each type of complication will significantly increase the risk of sarcopenia which is consistent with our findings.

In the age-based subgroup analysis (< 60 years group and ≥60 years group), the results indicated that the risk of sarcopenia in patients with diabetes aged < 60 years (OR: 3.21, 95% CI: 1.77—5.85) was higher than that in patients with diabetes that are aged ≥60 years (OR: 1.69, 95% CI: 1.03—2.78). However, this does not mean that as age increases, patients with diabetes have a lower risk of sarcopenia. First, age is a common risk factor for diabetes and sarcopenia. With age, insulin resistance becomes more common (9) and the ability of insulin to stimulate protein synthesis gradually weakens (42). Ageing is accompanied by major changes in body composition, including a gradual decrease in muscle mass, strength, and performance, which can markedly affect the daily activities of older adults (43, 44). Second, older age tends to be accompanied by a longer duration of diabetes, although this is not absolute. In a study from Japan on participants with diabetes aged ≥ 60 years (108 males and 105 females), the prevalence of sarcopenia was 19.2% and the risk of sarcopenia increased with longer duration of diabetes in women (OR = 1.43) (45). Finally, few studies on patients with diabetes aged <60 years were included, which may have induced bias.

The mechanisms involved in the association between diabetes and the risk of sarcopenia need to be fully elucidated, but some pathophysiological explanations have been provided. First, insulin resistance, through inhibition of the mTOR pathway (46), activation of autophagy (46), activation of the ubiquitin-proteasome proteolytic pathway (47), and accelerated muscle protein degradation may cause sarcopenia. Second, poor blood glucose control causes many metabolic abnormalities (10), such as the activation of apoptosis triggered by TNF-α (48) and impaired muscle mitochondrial oxidative capacity (49), thereby contributing to muscle cell damage (10). Third, repeated episodes of ischemia-reperfusion caused by vascular complications are a common pathway for muscle loss (50). Finally, in diabetic neuropathy, loss of motor neurons (51) or imbalance between denervation and reinnervation (51) can induce loss of muscle mass and strength.

In this study, some clinical issues were worthy of attention. In the prediabetes stage, it is crucial to reduce the risk of impaired glucose tolerance (IGT) progressing towards overt diabetes; therefore, lifestyle interventions are strongly recommended. In this regard, a clinical trial conducted in the U.S. (2.8 years of follow-up) showed that lifestyle intervention reduced the incidence of diabetes by 58% (95% CI: 48%—66%) in comparison with placebo (52). In addition, treatment with metformin and acarbose has a positive effect on reducing the risk of conversion from IGT to diabetes (52, 53). In patients with diabetes and sarcopenia, tight control of blood glucose levels is recommended, and for this reason, monitoring HbA1c levels and blood glucose monitoring should be strengthened.

The strength of this meta-analysis is that many factors associated with the risk of sarcopenia were considered, thus providing a more comprehensive analysis of the factors affecting sarcopenia. However, this study has certain limitations. First, due to the statistical difference in age and duration of diabetes between groups in some included studies, we cannot adequately consider these two variables, as they may cause bias in the results of this study. Second, data in some studies did not consider the influence of confounding factors, such as the severity of diabetes complications and the ranges of HbA1c and interactions among diabetes complications, which may have led to an overestimation of the study results. Third, although it had been meta-analyzed using multiple sarcopenia diagnostic criteria (AWGS, EWGSOP, FNIH criteria), the diagnostic criteria for sarcopenia also included observational studies measured by BIA. Fourth, the number of studies included in the subgroup analysis was relatively small, which may have caused false-negative results. Finally, the participants in many studies were not from the community but from the hospital, which may have resulted in lack of representativeness. In the future, more studies should be conducted to address these shortcomings.

In conclusion, high HbA1c levels, prediabetes, diabetes, and diabetes complications were significantly associated with an increased risk of sarcopenia. Whether these associations are further affected by age, sex and duration of diabetes complications needs to be tested in more prospective cohort studies. Meanwhile, therapeutic strategies aimed at avoiding the conversion of IGT to diabetes and optimizing glycemic control seem to be the best way to prevent or arrest sarcopenia in the diabetic population.

## Data Availability Statement

The original contributions presented in the study are included in the article/**Supplementary Material**. Further inquiries can be directed to the corresponding author.

## Ethics Statement

This article does not contain any studies with human participants or animals performed by any of the authors.

## Author Contributions

YQ contributed the interpretation of data, and drafting the report. ZZ contributed to statistical analysis, interpretation of data. YC and HG contributed the re-analysis, interpretation of data. J-BZ and RS contributed to study design and review. CS revised the manuscript. J-BZ is the guarantor of this work and, as such, had full access to all the data in the study and takes responsibility for the integrity of the data and the accuracy of the data analysis. All authors contributed to the article and approved the submitted version.

## Funding

This work was supported by the National Natural Science Foundation of China (No. 82070851, 81870556), Beijing Municipal Administration of Hospitals’ Youth Program (QML20170204), Excellent Talents in Dongcheng District of Beijing.

## Conflict of Interest

The authors declare that the research was conducted in the absence of any commercial or financial relationships that could be construed as a potential conflict of interest.

## Publisher’s Note

All claims expressed in this article are solely those of the authors and do not necessarily represent those of their affiliated organizations, or those of the publisher, the editors and the reviewers. Any product that may be evaluated in this article, or claim that may be made by its manufacturer, is not guaranteed or endorsed by the publisher.
