# Supplementary material for: The Association Between Diabetes Mellitus and Risk of Sarcopenia: Accumulated Evidences From Observational Studies
Source: Front Endocrinol (Lausanne). 2021 Dec 23;12:782391. doi: 10.3389/fendo.2021.782391 (PMC8734040; doi:10.3389/fendo.2021.782391)

**Supplement information**

**Search strategy**

**STable 1.** Risk of bias assessment (Newcastle-Ottawa Quality Assessment Scale criteria).

**SFigure 1**. Funnel plot of diabetes on risk of sarcopenia

**SFigure 2**. Funnel plot of diabetic complications on risk of sarcopenia

**Search strategy**

Pubmed：

(sarcopenia[MeSH] OR sarcopenia OR sarcopenias) AND ("Diabetes mellitus"[Mesh] OR diabetes OR "diabetes mellitus") Filters: Humans

714

Cochrane：

(sarcopenia OR sarcopenias) in All Text AND (diabetes OR “diabetes mellitus”) in All Text - (Word variations have been searched)

119

Embase：

(sarcopenia OR sarcopenias) AND (diabetes OR 'diabetes mellitus')

AND 'human'/de AND 'article'/it

821

Web of science：

((sarcopenia OR sarcopenias)) AND ((diabetes OR “diabetes mellitus”)) AND (human)

667

**STable 1.** Risk of bias assessment (Newcastle-Ottawa Quality Assessment Scale criteria).

| Study | Selection | | | | Comparability | Outcome | | | Quality  score |
| --- | --- | --- | --- | --- | --- | --- | --- | --- | --- |
|  | Representativeness of the exposed cohort | Selection of the non-exposed cohort | Ascertainment of exposure | Demonstration that the outcome of interest was not present at start of the study | Comparability of cohorts on the basis of the design or the analysis | Ascertainment of outcome | Was follow-up long enough for outcomes to occur? | Adequacy of follow-up of cohorts |  |
| Sanada(2010） | * | * | * | * | ** | * | * | * | 9 |
| Sugimoto(2019) | * | * | * | * | * | * | * | * | 8 |
| Yoon(2016） | * | * | * | * | ** | * | * | * | 9 |
| Sambashivaiah(2019) | * | 无 | 无 | * | * | * | * | * | 6 |
| Anbalagan(2013) | * | * | 无 | * | ** | * | * | * | 8 |
| Bouchi(2017) | * | * | 无 | * | ** | * | * | * | 8 |
| Kim(2014) | * | * | 无 | * | ** | * | * | * | 8 |
| Lim(2018) | * | * | 无 | * | ** | * | * | * | 8 |
| Mori(2019) | * | * | * | * | * | 无 | * | * | 7 |
| Souza(2019) | * | * | 无 | * | * | * | * | * | 7 |
| Wang(2016) | * | 无 | * | * | ** | * | * | * | 8 |
| Celiker(2018) | * | * | 无 | * | ** | * | * | * | 8 |
| Cheng(2017) | * | 无 | 无 | * | * | * | * | * | 6 |
| Fukuda(2017) | * | * | 无 | * | * | * | * | * | 7 |
| Yang(2020) | * | * | 无 | * | * | / | * | * | 7 |
| Yasemin(2019) | * | * | 无 | * | * | * | * | * | 7 |

**SFigure 1**. Funnel plot of diabetes on risk of sarcopenia


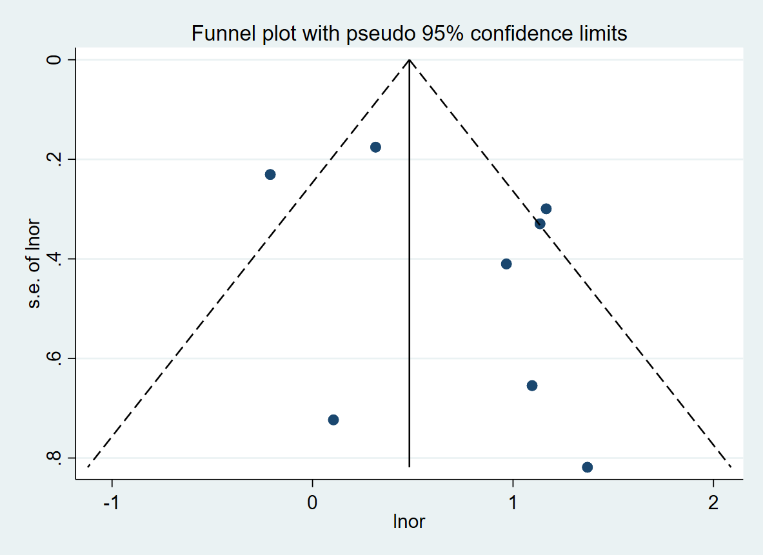


**SFigure 2**. Funnel plot of diabetic complications on risk of sarcopenia


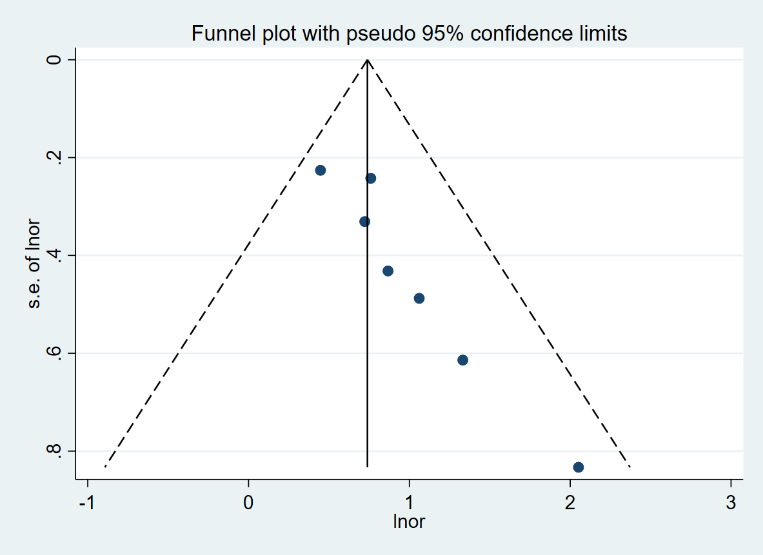

Supplement: Supplementary file 1 [file DataSheet_1.docx]
